# Cep192 Controls the Balance of Centrosome and Non-Centrosomal Microtubules during Interphase

**DOI:** 10.1371/journal.pone.0101001

**Published:** 2014-06-27

**Authors:** Brian P. O’Rourke, Maria Ana Gomez-Ferreria, Robin H. Berk, Alexandra M. U. Hackl, Matthew P. Nicholas, Sean C. O’Rourke, Laurence Pelletier, David J. Sharp

**Affiliations:** 1 Department of Physiology and Biophysics, Albert Einstein College of Medicine, Bronx, New York, United States of America; 2 Samuel Lunenfeld Research Institute, Mount Sinai Hospital, Toronto, Ontario, Canada; 3 Department of Anatomy and Structural Biology, Albert Einstein College of Medicine, Bronx, New York, United States of America; National Cancer Institute, NIH, United States of America

## Abstract

Cep192 is a centrosomal protein that contributes to the formation and function of the mitotic spindle in mammalian cells. Cep192’s mitotic activities stem largely from its role in the recruitment to the centrosome of numerous additional proteins such as gamma-tubulin and Pericentrin. Here, we examine Cep192’s function in interphase cells. Our data indicate that, as in mitosis, Cep192 stimulates the nucleation of centrosomal microtubules thereby regulating the morphology of interphase microtubule arrays. Interestingly, however, cells lacking Cep192 remain capable of generating normal levels of MTs as the loss of centrosomal microtubules is augmented by MT nucleation from other sites, most notably the Golgi apparatus. The depletion of Cep192 results in a significant decrease in the level of centrosome-associated gamma-tubulin, likely explaining its impact on centrosome microtubule nucleation. However, in stark contrast to mitosis, Cep192 appears to maintain an antagonistic relationship with Pericentrin at interphase centrosomes. Interphase cells depleted of Cep192 display significantly higher levels of centrosome-associated Pericentrin while overexpression of Cep192 reduces the levels of centrosomal Pericentrin. Conversely, depletion of Pericentrin results in elevated levels of centrosomal Cep192 and enhances microtubule nucleation at centrosomes, at least during interphase. Finally, we show that depletion of Cep192 negatively impacts cell motility and alters normal cell polarization. Our current working hypothesis is that the microtubule nucleating capacity of the interphase centrosome is determined by an antagonistic balance of Cep192, which promotes nucleation, and Pericentrin, which inhibits nucleation. This in turn determines the relative abundance of centrosomal and non-centrosomal microtubules that tune cell movement and shape.

## Introduction

Centrosomes are the primary microtubule (MT) nucleating and organizing centers in most eukaryotic cells and as such are important for numerous aspects of cellular development and function. Cells also contain additional populations of extra-centrosomal MTs, such as those derived from the Golgi apparatus, and a current belief is that shifts in the relative abundance of centrosome and non-centrosome-associated MTs impact cell morphogenesis, polarization and differentiation [1], [2], [3]. In general, centrosomal MTs are organized in a radial fashion with their dynamic plus-ends facing toward the cell periphery while non-centrosomal MTs populations can acquire a much more polarized distribution (e.g. Golgi-derived MTs orient toward the leading edge of motile cells) [4], [5], [6]. Thus, one can envision a scenario in which centrosomal and non-centrosomal MTs resist and promote cellular polarization, respectively.

The centrosome itself consists of two orthogonally positioned, barrel shaped centrioles surrounded by highly ordered rings of protein known as the pericentriolar material (PCM) [7], [8]. The PCM is composed of hundreds of proteins including MT nucleating factors, such as gamma-tubulin, MT anchoring proteins, such as ninein, scaffolding proteins, such as Pericentrin, and a variety of kinases and phosphatases, ubiquitinases, etc [9]. We and others have shown that Cep192 (centrosomal protein of 192 kDa) is an integral PCM component, which likely lies near the hierarchical base of the PCM assembly pathway [10], [11]. At the onset of mitosis, Cep192 becomes highly enriched at centrosomes (increasing roughly ten-fold relative to its interphase levels) and is at least partly required for the subsequent accumulation of gamma-tubulin, Pericentrin, Aurora A kinase and other PCM components. This process, known as centrosome activation, greatly enhances the centrosome’s MT nucleation capacity which in turn generates sufficient MTs for the formation of the MT-based spindle apparatus that separates chromosomes into the daughter cell products of cell division [10], [11], [12].

Though there is evidence suggesting that Cep192 is also important for the assembly and function of centrosomes in non-mitotic cells [10], [11], the interphase functions of this protein have not been examined as thoroughly. Here we show that Cep192 remains fundamentally involved in the formation and function of interphase centrosomes. Depletion of the protein from interphase cells results in a significant reduction in centrosomal MT nucleation but enhances MT nucleation from other sites, including the Golgi apparatus. Our data also indicate that although Cep192 continues to play important roles in regulating the composition of interphase centrosomes, it does so in ways that are clearly distinct from mitosis. This is particularly notable with regard to Pericentrin, which appears to antagonize Cep192’s recruitment to centrosomes and also to reduce centrosomal MT nucleation. Finally, cells depleted of Cep192 acquire hyperpolarized morphologies, supporting the aforementioned hypothesis that centrosomal MTs resist cell polarization, and lose the ability to undergo efficient migration.

## Materials and Methods

### Antibodies

Our human Cep192 antibody was generated in rabbit against a specific peptide sequence (MKTSDLVPSFGYFIRSPEKREPC) (Proteintech). Cep192 antibody was applied at 1∶2000 (1.5 µg/ml) in blocking buffer (5% normal goat serum in PBS with 0.1% Triton X-100) and at 1∶2000 in Western Blots. Cep215 antibody was a gift of Dr. Kunsoo Rhee (Seoul National University) and ALMS1 antibody a gift of Dr. David Wilson (University of Southampton). Other commercially available primary antibodies used were mouse monoclonal anti-α-tubulin (Sigma-Aldrich, DM1A, T9026), mouse monoclonal anti-γ-tubulin (GTU88, Sigma-Aldrich, T6557), rabbit polyclonal anti-α-tubulin (Abcam, ab18251), mouse monoclonal anti-Centrin (Millipore, 04-1624), rabbit monoclonal GAPDH (Novus Biologicals, NB100-79955), mouse monoclonal anti-acetylated tubulin (Sigma, T6793), mouse monoclonal anti-FLAG (Sigma, F1804) and mouse monoclonal anti-tyrosinated tubulin (Sigma, T9028). Secondary antibodies conjugated with the fluorophores (Jackson ImmunoResearch Laboratories) were used at a final concentration of 7.5 µg/ml.

### Cell Culture

U2OS (ATCC), U2OS EB1-GFP ([13]), and WM-266-4 (ATCC) cells were cultured at 37°C in DMEM (Invitrogen, 11965-092) containing 10% fetal-bovine serum, 1% Glutamax, 2% penicillin/streptomycin in the presence of 5% CO_2_. RPE1 Centrin-GFP cells ([14]) were cultured in DMEM/F-12 (1∶1) medium (Corning, 10-090-CV) supplemented with 10% FBS and 1% penicillin-streptomycin. Low passage HEKa cells were obtained from Invitrogen (C-005-5C) and cultured in Epilife Medium (Invitrogen, M-EPI-500-CA) with EpiLife Defined Growth Supplement (S-012-5).

Cep192 inducible expression cell line was generated using the Flp-In T-REx system (Invitrogen). Cep192-2 cDNA was cloned in the pcDNA5-FRT-TO-FLAG vector previously described [15]. This construct and the pOG44 vector (expressing FLP recombinase) were co-transfected into the U2OS Flp-In T-REx host cell line. Cells that had integrated the expression plasmid were selected with Hygromycin B and Blasticidin for 2–3 weeks. Individual colonies were expanded and analyzed.

### siRNA Transfection and Induced Expression

To knockdown Cep192, 1.5 µl of 20 nM double-stranded siRNA nucleotides targeting either regions conserved in all Cep192 isoforms (pan-Cep192) (5′-CACAUGAUGCCUGCUAGUU-3′, 5′-GACACUUUCUUCAUGAGCA-3′, 5′-GGACUUAAGUGCUACUAGU-3′) or specifically the N-terminal region of the Cep192-1 isoform (5′-GCUUAAACUGCAAGUUUCAAUCAGA-3′) were transfected with RNAiMax (Invitrogen, 13778075) following manufacturer’s protocol. The Cep192-1 isoform targeting sequence was used in rescue experiments; pan-Cep192 siRNA was used in all other experiments. Scrambled siRNA was used as a negative control (Sigma-Aldrich, SIC001). Depletion of Pericentrin was completed following previously published protocols (5′-GCAGCUGAGCUGAAGGAGATT-3′) [16]. Efficiency of knockdown was measured both by quantitative immunofluorescense and by Western Blot analysis. Samples were suspended in Laemmli buffer and separated through 10% sodium dodecyl sulfate-polyacrylamide gel electrophoresis (SDS-PAGE) and transferred onto nitrocellulose membranes.

For rescue experiments, cells were depleted of Cep192 with the Cep192-1 isoform specific siRNA. 48 hours after transfection, cells were induced with 1 µg/mL of tetracycline for 24 hours to express FLAG-Cep192-2 to endogenous levels. Protein expression was quantified with Western Blot and visualized localizing to the centrosome by immunofluorescence. This treatment condition is referred to as “Cep192 siRNA-Induced”.

### Immunofluorescence

Cells were grown on coverslips and fixed in either 100% methanol at −20°C for 20 min or 4% paraformaldehyde (PFA), 0.15% gluteraldehyde, 0.1% Triton X-100 in BRB-80 at room temperature for 15 min. Methanol fixed cells were rehydrated with PBS-Tween and blocked in buffer (5% normal goat serum in PBS with 0.1% Triton X-100). PFA fixed cells were rinsed with PBS then quenched with sodium borohydrite for 15 min 2X, rinsed in PBS again and then processed. Fixed cells were then incubated with the indicated primary antibody for 1.5 hours, washed with PBS-T 3×5 min, then incubated with fluorophore conjugated secondary antibody for 45 minutes. Cells were then washed again 3×5 min with PBS-T and mounted on a slide with mounting media.

### Microscopy

Fixed and live cells were imaged using a 4-D spinning-disk confocal microscope (PerkinElmer) with either a 10×(0.3 NA), 20×(0.5 NA), 60×(1.4 NA), or a 100×(1.4 NA) objective attached to a digital camera (Orca ER; Hamamatsu). Confocal images are displayed as the maximum intensity projections of all captured Z planes.

To characterize MT dynamics following Cep192 knockdown, three minute movies capturing EB1-GFP comet dynamics every 3 seconds were made following 72 hours of siRNA treatment. We utilized PlusTipTracker to analyze the following parameters for all movies in the dataset: Maximum gap length, 5 frames; minimum track length, 3 frames; search radius range, 8–12 pixels; maximum forward angle, 30°; maximum backward angle, 10°; shrinkage factor, 1.5; fluctuation radius, 1 pixel, consistent with EB protein tracking done in U2OS cells [17]. Data were exported to Excel (Microsoft) and analyzed using GraphPad Prism (GraphPad Software).

Wound healing assays were performed using the Ibidi culture insert dishes (Ibidi, 80206). After 48 hours of treatment, inserts were pulled between confluent wells, and cells migrated to close the vacant wound zone. Time lapse phase-contrast images of cells were acquired every 15 minutes using a 37°C heated chamber with 5% CO_2_ and humidity control. Cell migration closure efficiency was quantified by hand tracking the leading edge of the cells over time and comparing uncovered area in the zone after initial wounding relative to area remaining after closure by controls.

### Image Analysis

To comparatively quantify fluorescence intensity, data sets were acquired under identical conditions between control and experimental treated cells. Centrosomal fluorescence intensity was quantified by identifying cells with two centrin dots, creating a uniform, consistent region of interest large enough to fully encapsulate the corresponding PCM, and then measuring the intensity using ImageJ (NIH). Mean tubulin intensity was calculated by encircling each cell and measuring tubulin fluorescence intensity with Image J. Following background subtraction, the mean intensities were compared to controls and graphed using GraphPad Prism (GraphPad Software).

To compare MT regrowth at the centrosome, U2OS cells were plated onto coverslips and treated according to experimental conditions. Following 72 hours of treatment, cells were placed on ice for 40 minutes. MTs were regrown with 30 seconds of incubation in 37°C media. Cells were then fixed and stained with EB1 and Cep192 antibodies. MT tracks were directly counted by hand using ImageJ software.

To quantify extra-centrosomal MT regrowth, RPE1 cells were plated on coverslips, depleted of siRNA for 72 hours and processed as described elsewhere, allowing 25 seconds of regrowth in 21°C media and 2 minutes in extraction buffer before fixation in methanol and immunostaining [18], [19]. Extra-centrosomal MTs were counted by hand using ImageJ software.

### Statistical Analysis

Differences between treatments were analyzed using a nonparametric *t*-test (student’s t-test) for two-group comparisons with GraphPad Prism (GraphPad Software). S.E.M. indicates standard error of the mean. S.D. indicates standard deviation. Measurement means were taken to be statistically different if P<0.05. Minimal datasets available in Dataset S1.

## Results and Discussion

### Cep192 regulates interphase MT organization and dynamics

**Figure 1:**
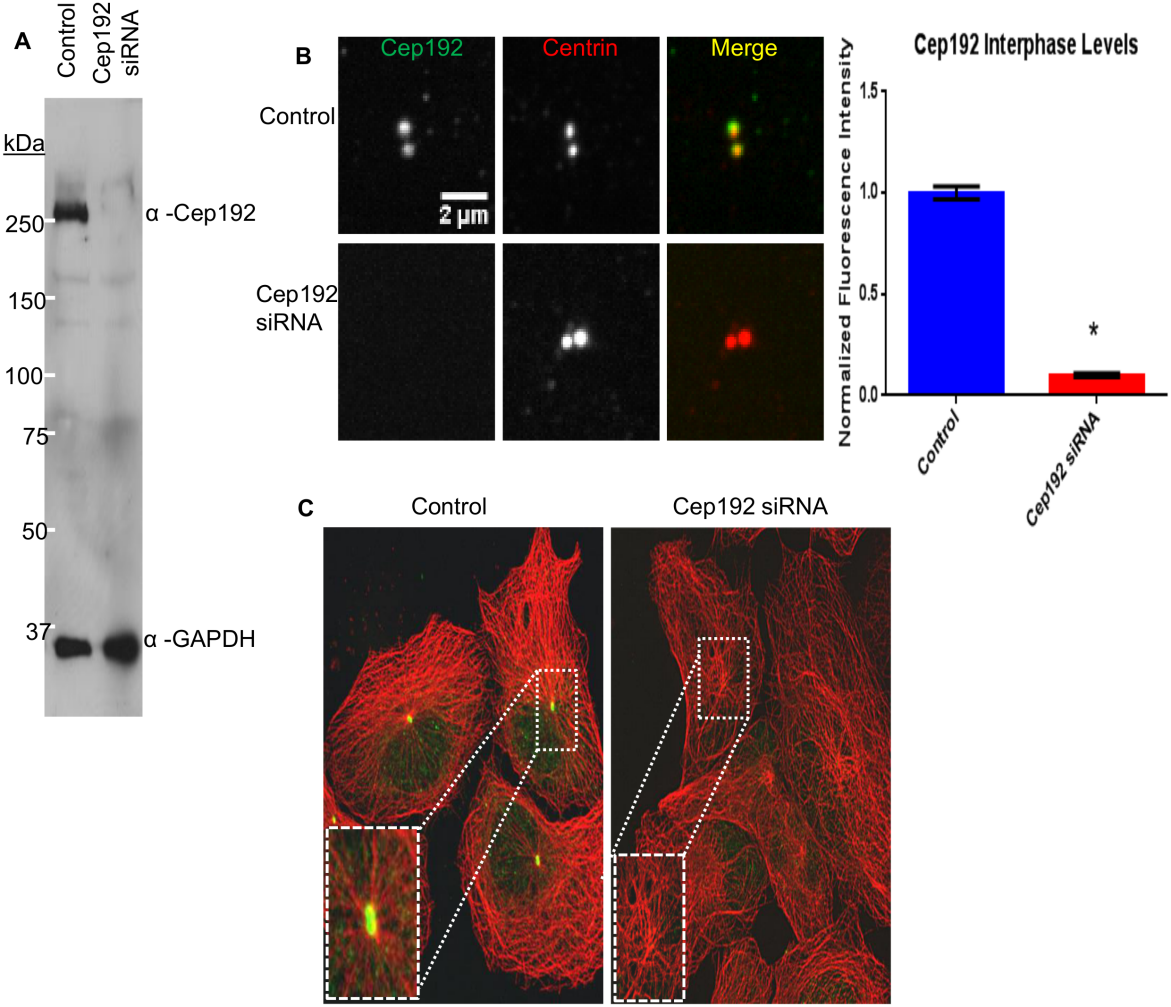
Optimized siRNA-mediated knockdown of Cep192. A) Western blot of control and Cep192 siRNA-treated U2OS cell lysates probed with anti-Cep192 and GAPDH (loading control) antibodies. Densitometry measurements using ImageJ indicated a significant (98%) decrease in Cep192 after siRNA treatment; P<0.0001. Values quantified from 3 separate blots. B) Quantitative immunofluorescence analysis revealed a >90% decrease in centrosome-associated Cep192 staining after siRNA treatment. Vertical bars represent S.E.M. P value <0.0001. N≥37 centrosomes per experiment from 3 independent experiments. C) Immunofluorescence micrographs of control and Cep192 siRNA-treated U2OS cells (72 hours after siRNA treatment) double-labeled for Cep192 (green) and MTs (red). Controls showed robust radial arrays of centrosome-associated MTs arrays while siRNA-treated cells were devoid of Cep192 staining and displayed predominantly non-radial MT arrangements.

Though there is widespread agreement that Cep192 is essential for centrosome assembly and function in mitosis, analyses of the protein’s role in interphase have been limited, with conflicting results reported in different studies [10], [11]. Evaluation of these earlier studies–which have all relied on RNAi-mediated depletion of Cep192–is hampered by the variable and incomplete levels of protein knockdown achieved in each. Thus, the first goal of the current study was to identify optimal conditions for siRNA (small interfering RNA) mediated knockdown (KD) of Cep192 in interphase cells. We were able to achieve nearly complete (>95%) knockdown of Cep192 in human U2OS cells within 72 hours after transfection of a mixture of three different siRNAs using the RNAiMax transfection reagent (InVitrogen). The extent of Cep192 knockdown was determined in bulk using Western blotting and at the level of individual centrosomes using quantitative immunofluorescence (Fig. 1A–C). Similar results were obtained in a wide variety of cell types including several cancer lines, primary human epidermal keratinocytes (HEKa) (Invitrogen) and Centrin-GFP RPE1 cells.

Having thus established optimal conditions for knocking down Cep192, we set out to determine whether/how the loss of Cep192 impacts the gross morphology of steady state interphase MT arrays using cultured U2OS cells as a model. U2OS is an osteosarcoma cell line which acquires an adherent epithelial morphology in culture [20]. The cytoskeleton of these cells is easy to image using fluorescence microscopy and, for the purposes here, we utilized our in-house spinning disk confocal microscope to obtain complete z-series of methanol-fixed U2OS cells double-labeled for α-tubulin and the centriolar protein, centrin, which unambiguously marks the centrosome [10], [21], [22], [23].

**Figure 2:**
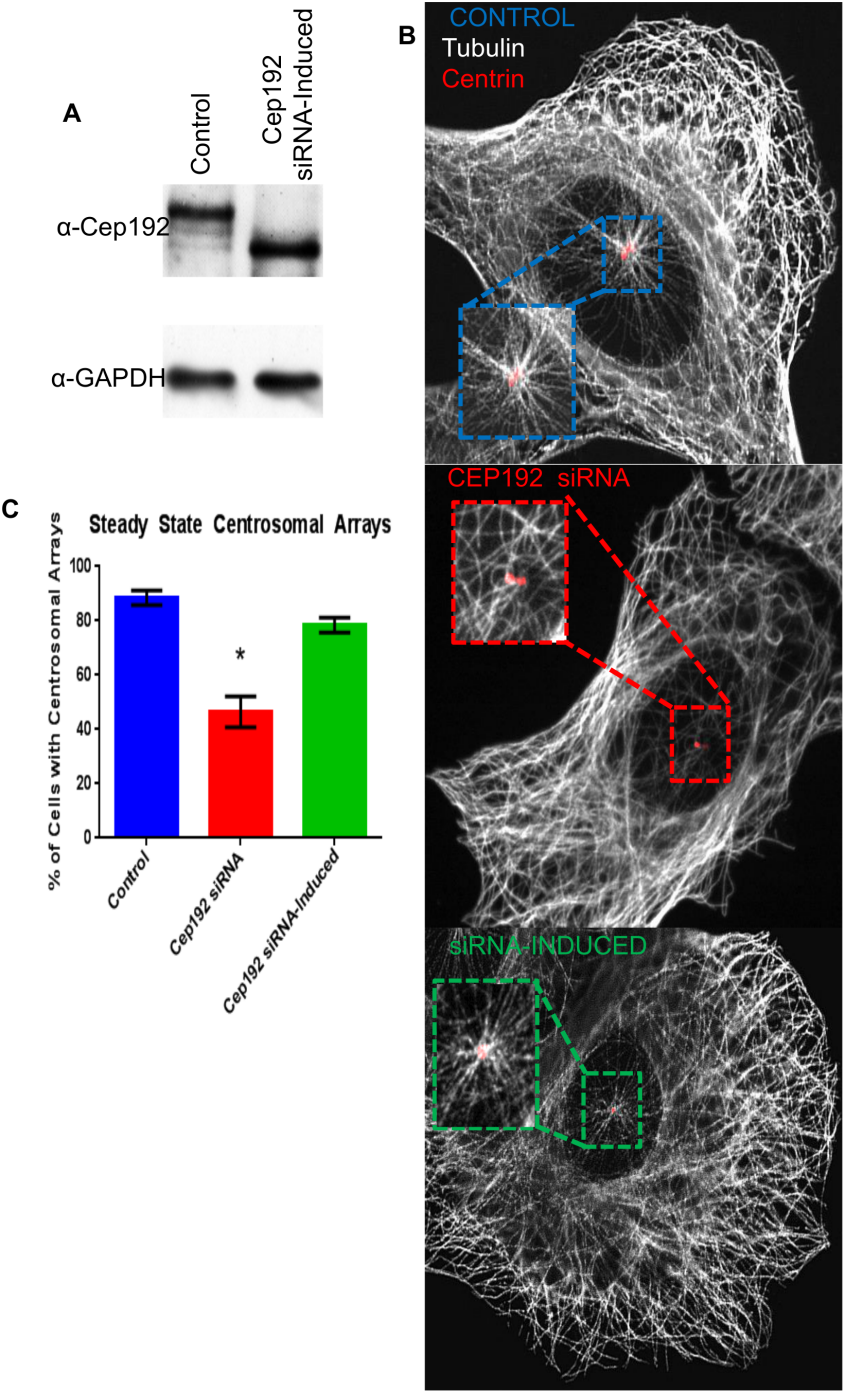
Inducible Cep192 expression rescues centrosomal arrays in FLAG-Cep192-2 Flp-In T-Rex U2OS cells. A) Cep192 was successfully depleted using siRNA targeted specifically to the Cep192-1 isoform. Using an inducible U2OS tetracylcine inducible FLAG-Cep192-2 cell line, the shorter exogenous protein, resistant to the siRNA, is expressed at wild type levels. Following normalization to GAPDH, Cep192-2 levels were within 6.6% of controls. B) Representative immunofluorescence micrographs of MT arrangement in each experimental condition. Boxed and magnified regions focus on centrin labeled sites. C) Graph showing the percentage of cells displaying obvious centrosome MT arrays in each condition (24 hours after Cep192-2 induction; 88.5% for control, 46.5% for Cep192 KD, and 78.4% for knockdown induced cells). Vertical bars represent S.D. P<0.0001. N≥10 cells per condition from 3 independent experiments.

In the vast majority of control-treated interphase U2OS cells, robust astral arrays of centrosome-associated MTs were clearly visible on top of or adjacent to the nucleus and these were easily distinguishable from the numerous non-centrosome-associated MTs positioned elsewhere in the cell (Fig. 2A) [24]. By contrast, obvious centrosome-associated MT asters were absent in more than half of the Cep192 depleted cells analyzed with many of the remainder displaying a qualitative reduction in the size of their centrosomal MT arrays (Fig. 2B, C). This phenotype was rescued by the induced expression of a FLAG-tagged Cep192-2 construct; Cep192-2 is a short splice variant not normally expressed in U2OS cells and is resistant to the siRNA treatment used in this assay–this siRNA recognizes the mRNA sequence that is specific to the larger Cep192-1 isoform predominant in U2OS cells[25]. FLAG-tagged Cep192-2 localizes to centrosomes similarly to the endogenous Cep192-1 protein (Fig. S2).

**Figure 3:**
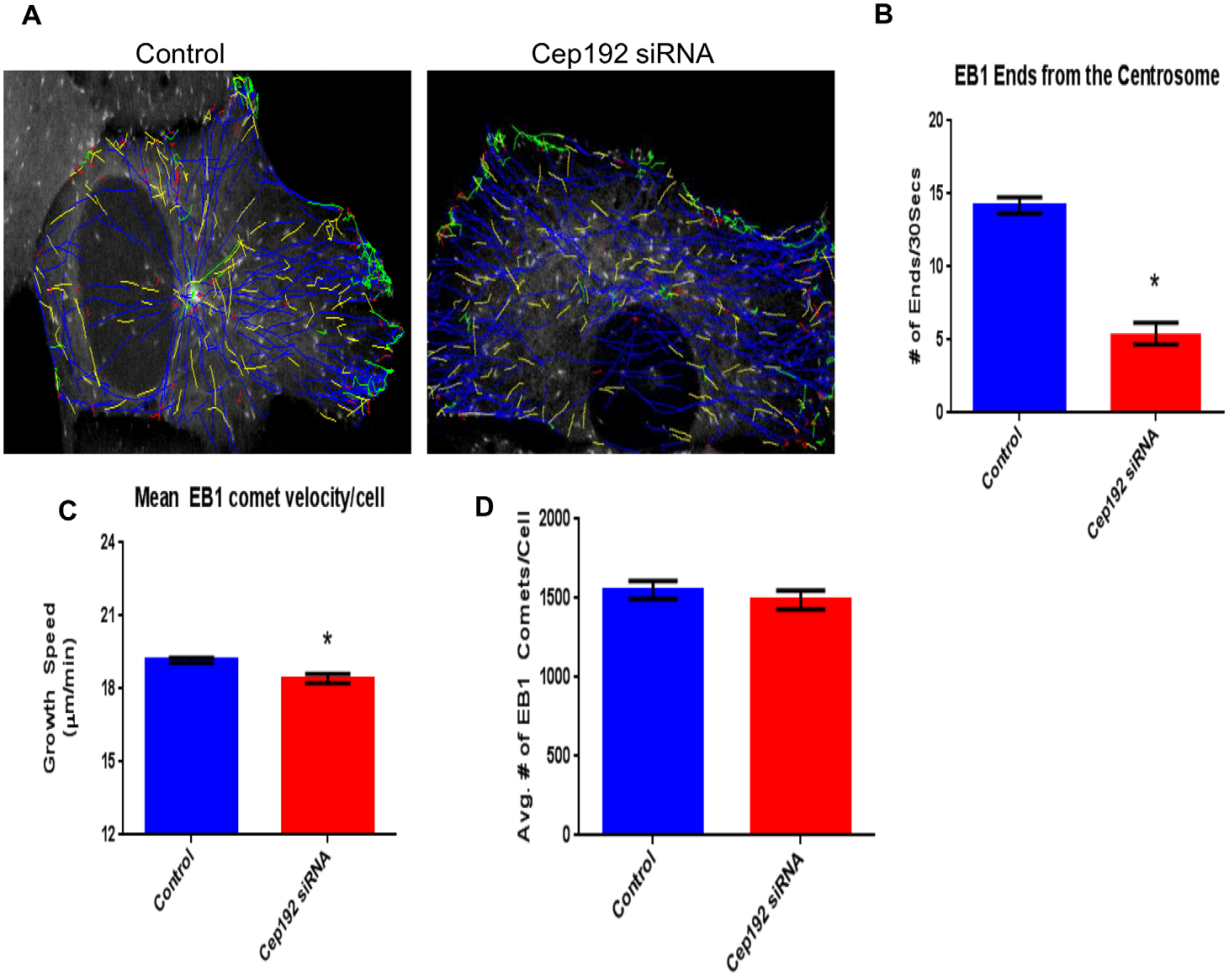
Cep192 is required for normal MT dynamics. A) Representative images of tracked EB1-GFP comet trajectories during a 30s time interval in control and KD conditions in U2OS cells. Note the lack of a major MT nucleating site in the Cep192 KD cell. B) MT tracks emanate from the centrosome significantly less frequently in Cep192 KD cells relative to control (5.4 comets/centrosome vs. 14.2 (comets/centrosome)). C) Using the automated tracking program, plusTipTracker to track EB1 comets, we found comets in Cep192 depleted cells traveled at a significantly slower rate relative to control (18.4±0.193 µm/min vs. 19.2±0.112µm/min, respectively). D) Despite changes in MT organization, there was no change in the number of comets per cell. Vertical bars represent S.E.M. P values are <0.0001, P = 0.0007, and P = 0.4377, respectively. N≥16 cells per experiment from 3 independent experiments per condition.

As a complement to our analyses of fixed cells, we used 4-D spinning disk confocal microscopy to examine Cep192’s impact on interphase MT organization and dynamics in living U2OS cells expressing EB1-GFP (Movie S1, Movie S2). EB1-GFP selectively associates with the plus-ends of polymerizing MTs, thus appearing as comets that move through the cytoplasm, and its behaviors can be automatically tracked using PlusTipTracker software to quantitatively identify MT growth patterns within the context of the cell [17], [26], [27]. Figure 3A compares representative EB1-GFP comet trajectory paths obtained from interphase control and Cep192 siRNA treated cells. In controls, MT growth trajectories displayed a dominantly radial pattern, emerging from a single perinuclear locus, which is most likely the centrosome. We further found that EB1-GFP labeled MT plus-ends emerged from this locus at an average rate of 28.42±1.1 ends/minute (Fig. 3B). In contrast, radial MT growth patterns were much less prominent after the depletion of Cep192 and the rate of EB1-GFP comet emergence from the center of observable asters was significantly reduced ∼3-fold relative to controls (10.86±1.509 ends/minute). These data, in concert with our immunofluorescence analyses, clearly show that Cep192 normally influences the steady-state MT array of interphase cells by promoting the formation of centrosomal MTs. As an aside, tracking of EB1-GFP comet movements also indicated a small but highly reproducible and statistically significant decrease in MT plus-end growth speed in Cep192 siRNA treated cells relative to controls (18.42±0.193 µm/min in Cep192 siRNA treated cells vs. 19.18±0.112 µm/min in controls; Fig. 3C).

**Figure 4:**
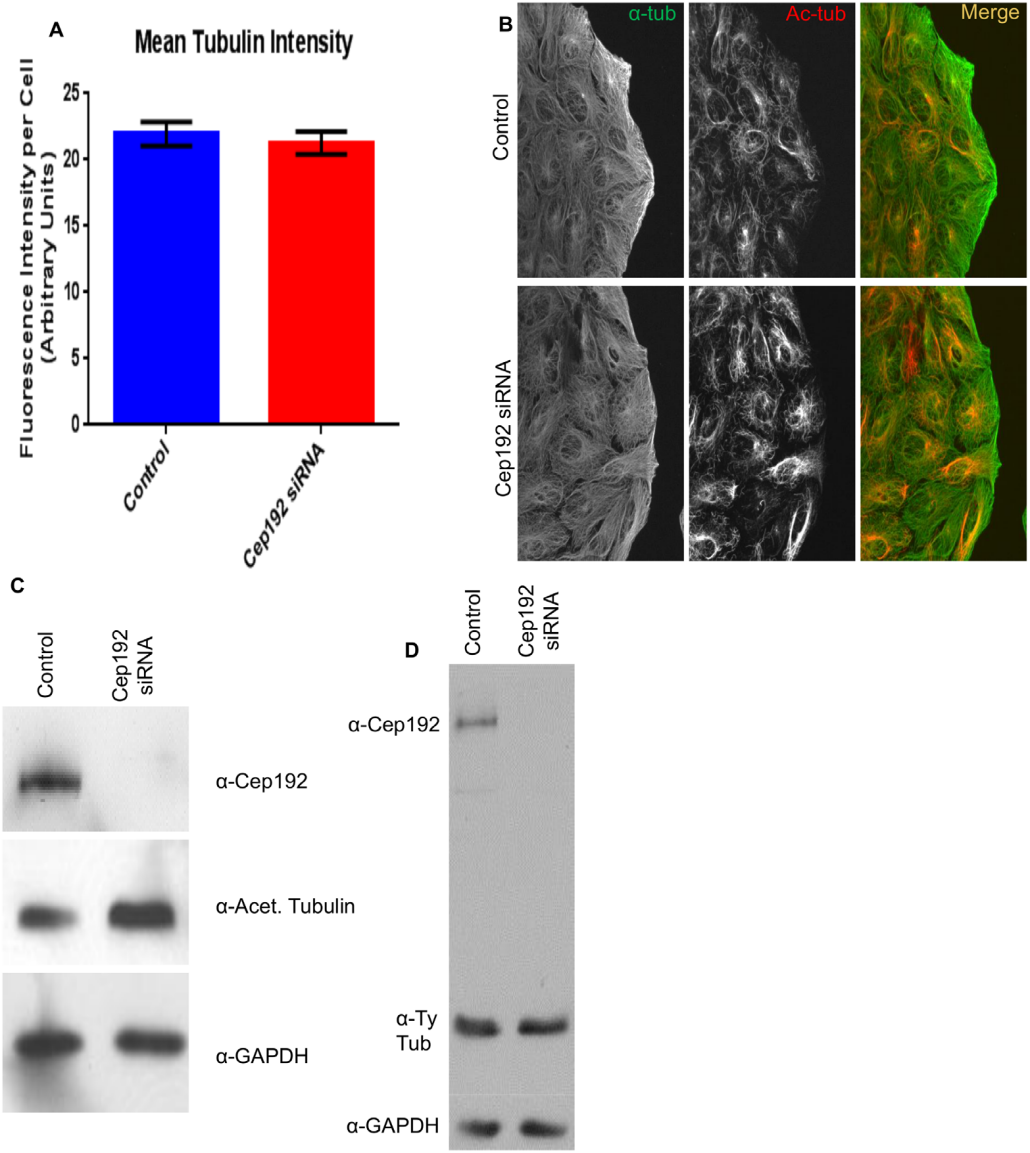
Cep192 knockdown causes a positive shift in acetylated tubulin density in U2OS cells. A) Quantitative immunofluorescence of cells immunostained for alpha-tubulin showed no significant shift in total interphase MT polymer mass between control and Cep192 depleted cells. Vertical bars represent S.E.M. P value is 0.5951 N≥20 cells per experiment from 3 independent experiments. B) Immunofluorescence micrographs showing control and Cep192 siRNA-treated U2OS cells double labeled for acetylated tubulin and α-tubulin. C) Western blot control and Cep192 siRNA-treated U2OS cell lysates stained for acetylated tubulin. Densitometry measurements indicated that acetylated tubulin increases ∼64% following Cep192 knockdown. GAPDH is shown as a loading control. D) Levels of tyrosinated tubulin decrease 9% following Cep192 KD.

Surprisingly, despite the apparent decrease in the formation of centrosomal MTs following Cep192 depletion, the average total number of EB1-GFP comets in control vs. Cep192 siRNA treated cells was statistically indistinguishable (1577±160.4 comets/cell in controls vs. 1486±65.8 comets/cell in Cep192 siRNA treated cells; Fig. 3D). Quantitative immunofluorescence of cells stained for MTs also revealed no significant shift in the total interphase MT polymer mass after Cep192 siRNA treatment (Fig. 4A). It is notable, however, that the MTs that form in the absence of Cep192 were found to be quite different in terms of the post-translational modifications acquired by their tubulin subunits. In particular, both quantitative immunofluorescence and Western blotting revealed a significant ∼60% increase in the levels of longer-lived, less dynamic acetylated MTs as well as a smaller but also significant ∼10% decrease in shorter-lived, more dynamic tyrosinated MTs (Fig. 4B, C, D) [28], [29], [30].

**Figure 5:**
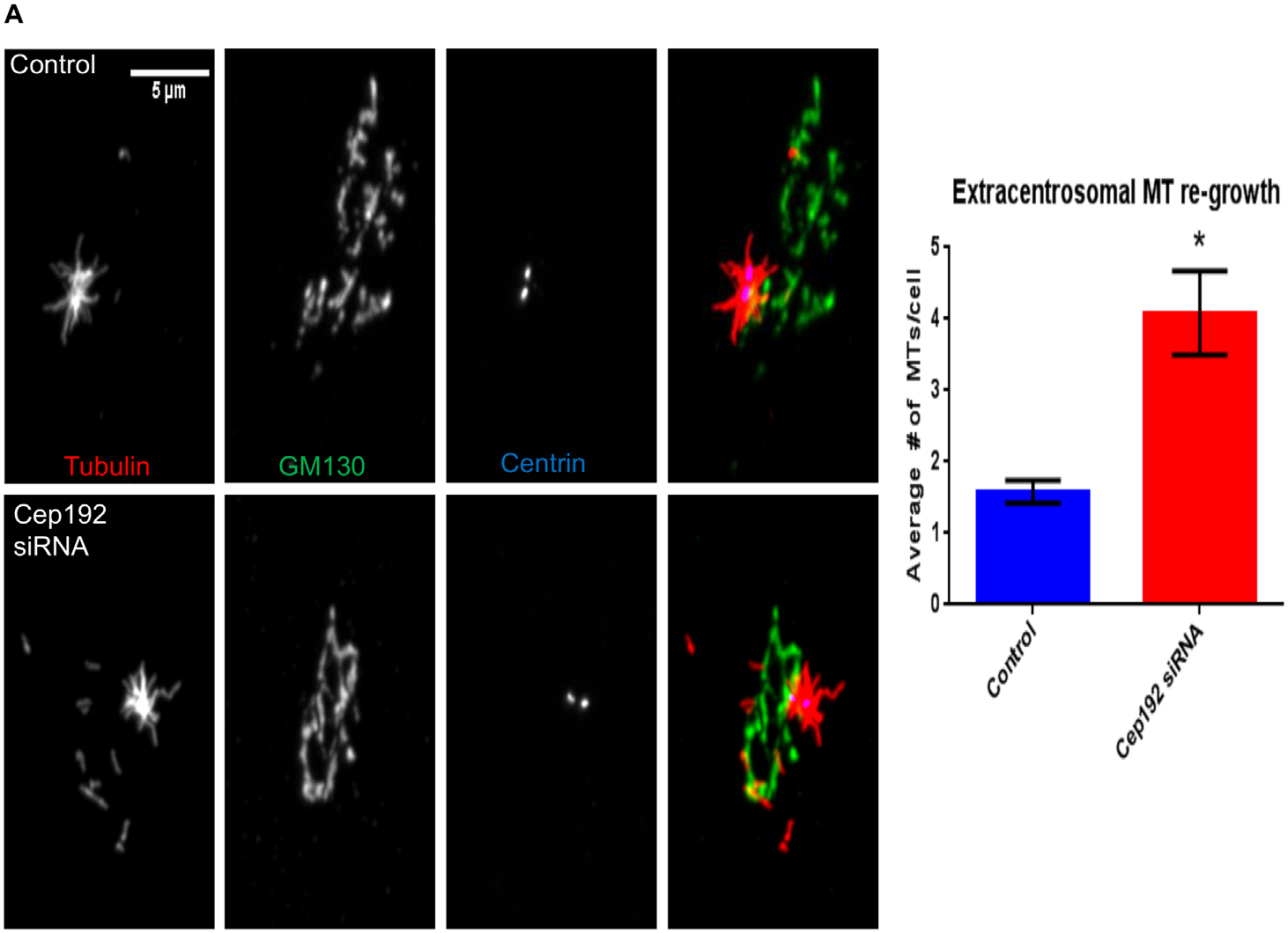
Cep192 depletion increases extra-centrosomal MT growth in RPE1 Centrin-GFP cells. A) MT regrowth after ice depolymerization was used to quantify the relative proportion of MTs nucleated from centrosomal and extra-centrosomal sites. The images in this panel are immunofluorescence micrographs of control and Cep192 siRNA treated RPE1 triple-labeled for MTs, the Golgi marker GM130, and Centrin (Centrin is GFP-tagged). After 25 seconds of MT regrowth, control cells contained an average of 1.57 MTs/cell non-centrosomal MTs (neither end attached to the centrosome), while Cep192 depleted cells contained an average of 4.05 non-centrosomal MTs. Vertical bars represent S.E.M. P = 0.0002. N≥28 cells per experiment from 3 independent experiments.

If cells lacking Cep192 produce fewer centrosomal MTs, how are they then capable of maintaining an overall MT polymer mass on par with controls? One obvious possibility is that the loss of centrosomal MTs following Cep192 knockdown is augmented by increased MT nucleation at non-centrosomal loci such as the Golgi apparatus, which has recently been identified as a prominent secondary MT organizing center important for cell polarization and motility [4], [5]. Indeed, in some cell types, such as human RPE1 (retinal pigment epithelial cells), the Golgi apparatus produces MTs at a level that is roughly equivalent to the centrosome [5]. Golgi-derived MTs also become highly acetylated relative to their centrosomal counterparts [3]. We initially attempted to test whether the depletion of Cep192 results in increased MT nucleation from the Golgi apparatus in U2OS cells using a MT regrowth assay–in this assay, total cellular MTs are cold depolymerized by ice treatment, then re-polymerized when cells are shifted to room temperature. Unfortunately, this proved to be impossible owing to the severe fragmentation of the Golgi apparatus occurring when MTs are depolymerized in this cell line. However, this line of analysis was possible in RPE1 cells which maintain a relatively organized Golgi apparatus even in the total absence of MTs [18]. After 25 seconds of MT regrowth, Cep192 siRNA treated RPE1 Centrin-GFP cells displayed a 2.6-fold relative increase in the number of non-centrosomal MTs (neither end attached to the centrosome) with the majority of these having one end clearly associated with the Golgi apparatus (Fig. 5). Thus, we propose that a major function of Cep192 is to control the balance of centrosome and non-centrosome associated MTs. Given the results of previous studies, it seems most likely that Cep192 does so by recruiting gamma-tubulin and other MT nucleating factors to the centrosome [5].

### Cep192 and Pericentrin antagonistically control the extent of MT nucleation from interphase centrosomes

**Figure 6:**
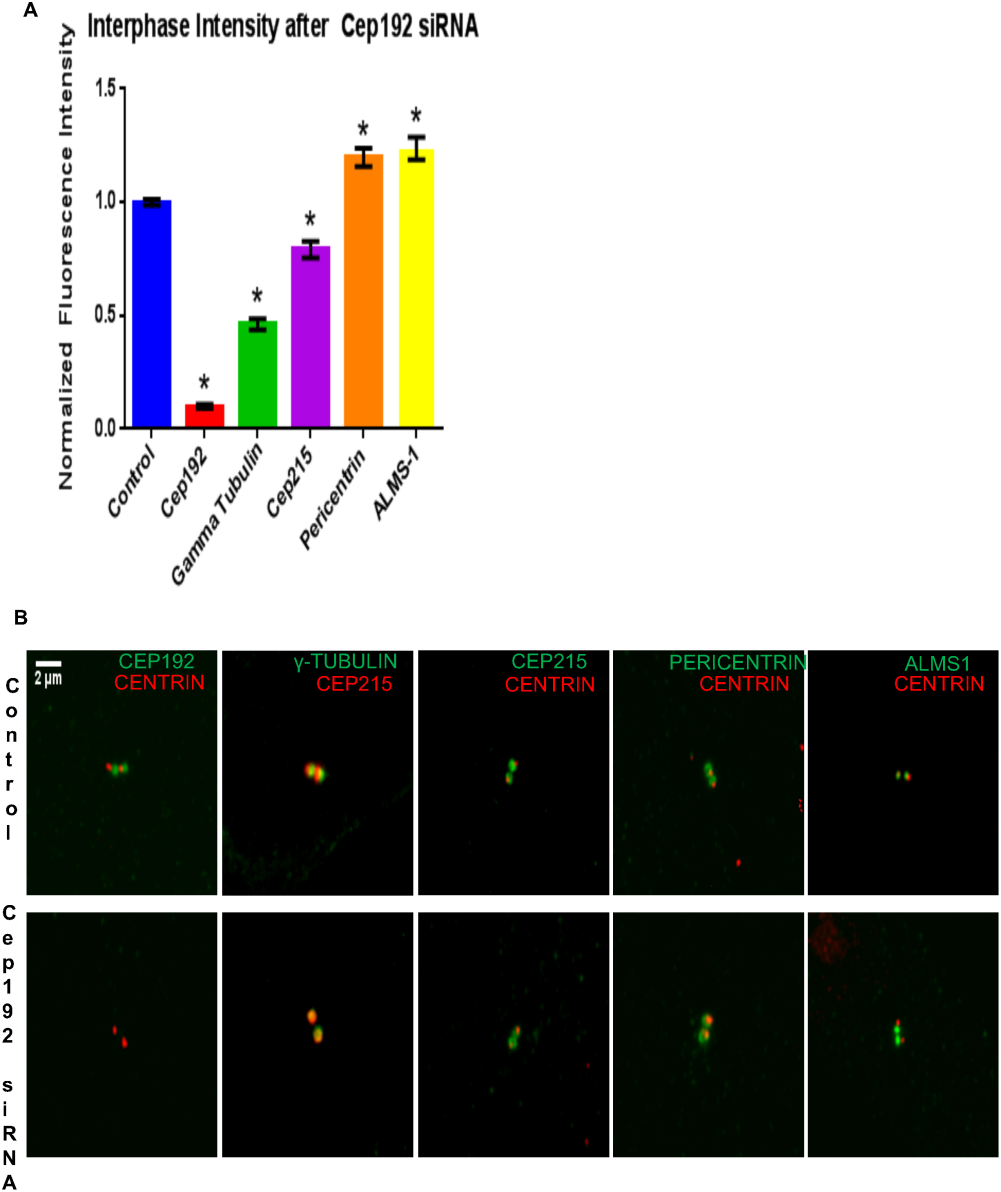
Cep192 is required for the assembly of the interphase centrosome. A) Graph quantifying the effects of Cep192 siRNA on the levels of centrosome-associated PCM proteins as determined by quantitative immunofluorescence. P values for all experiments are ≤0.0003. S.E.M. is depicted as vertical bars. N≥23 cells per experiment from 3 independent experiments. B) Representative immunofluorescence micrographs showing the alterations in PCM protein staining levels quantified in A.

To better understand the mechanism by which Cep192 stimulates MT growth from interphase centrosomes, we performed quantitative immunofluorescence analyses comparing the centrosomal levels of several PCM components in control vs. Cep192 siRNA-treated cells. Consistent with several previous reports, we found that the depletion of Cep192 significantly reduced the interphase levels of centrosome-associated gamma-tubulin ∼50% relative to controls, which likely directly accounts for the attenuated nucleation of centrosomal MTs observed after Cep192 siRNA treatment (Fig. 6) [11]. The depletion of Cep192 also reduced the level of centrosome-associated Cep215, another putative PCM scaffolding protein involved in the mitotic recruitment of centrosomal gamma-tubulin, albeit to a substantially lesser extent than gamma-tubulin (Fig. 6) [31].

**Figure 7:**
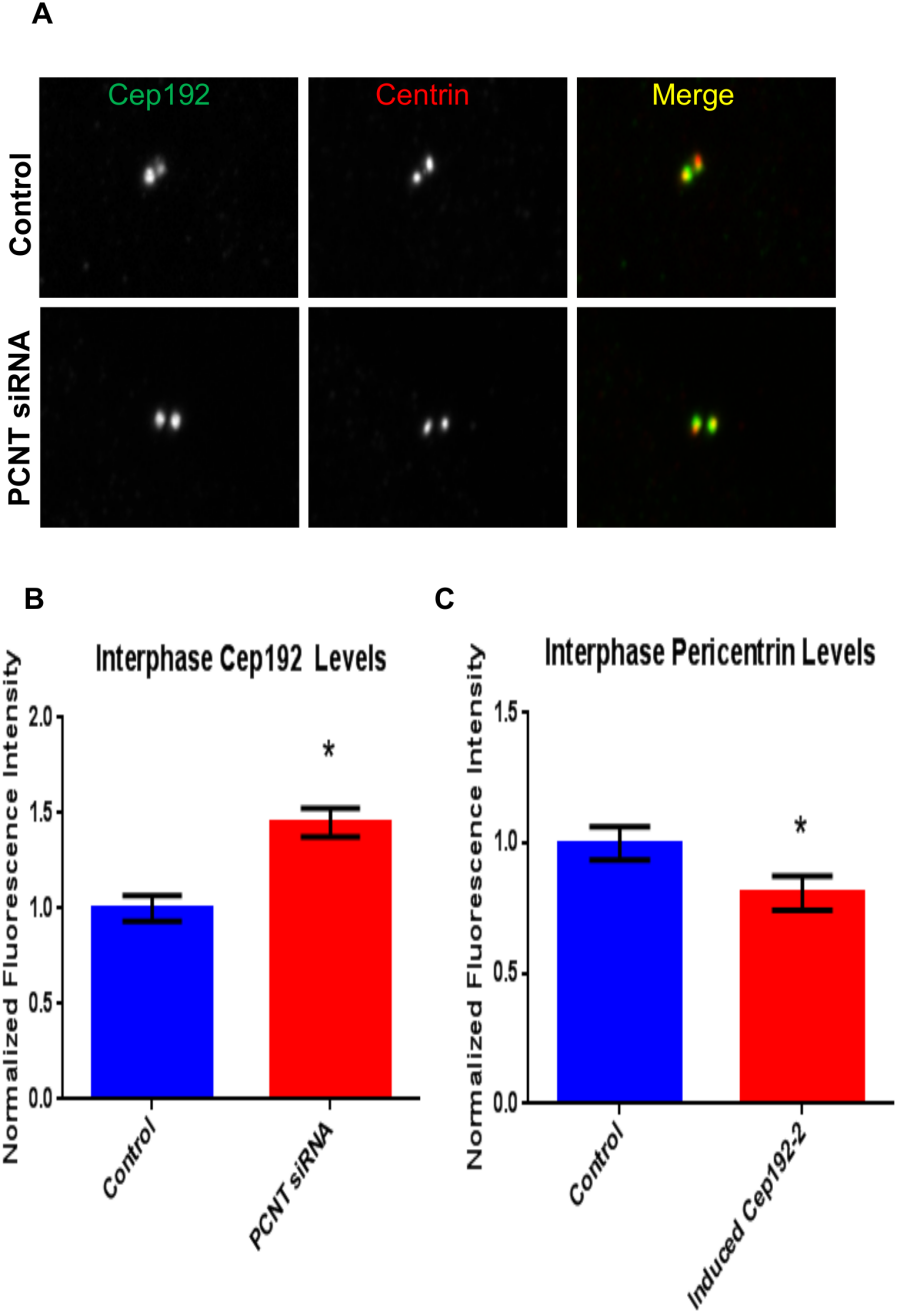
Altering Pericentrin levels impacts Cep192 localization to the centrosome and vice versa. A, B) Following 48 hours of siRNA depletion, interphase centrosomes showed an increase of 1.45X of Cep192. Vertical bars represent S.E.M. P value is <0.0001. N≥13 cells per experiment from 3 independent experiments. C) Overexpression of FLAG-Cep192-2 in U2OS cells resulted in a 20% decrease in Pericentrin localization to centrosomes relative to control. Vertical bars represent S.E.M. P = 0.0389. N≥16 cells per experiment from 3 independent experiments.

In stark contrast, we found that Cep192 knockdown significantly increased the levels of interphase centrosome-associated Pericentrin–as well as a second centrosome protein termed ALMS1, which has also been shown to interact with Cep192 (Fig. 6) [32]–while overexpression of FLAG-Cep192-2 reduced Pericentrin levels at interphase centrosomes by ∼20% (Fig. 7). The converse was also true as levels of centrosome-associated Cep192 were significantly increased in interphase cells depleted of Pericentrin (Fig. 7A–C). These data indicate a situation quite different from mitosis, when the depletion of either protein results in decreased centrosomal localization of the other, and instead raise the intriguing possibility Pericentrin actually functionally antagonizes Cep192 during interphase. Indeed, the relative abundance of these proteins could serve as a means to control the rates of centrosomal MT nucleation. Additional support for this hypothesis comes from recently published work showing that the Pericentrin orthologue Pericentrin-like Protein (PLP) negatively regulates centrosomal activity in *Drosophila melanogaster* neuroblasts [33].

**Figure 8:**
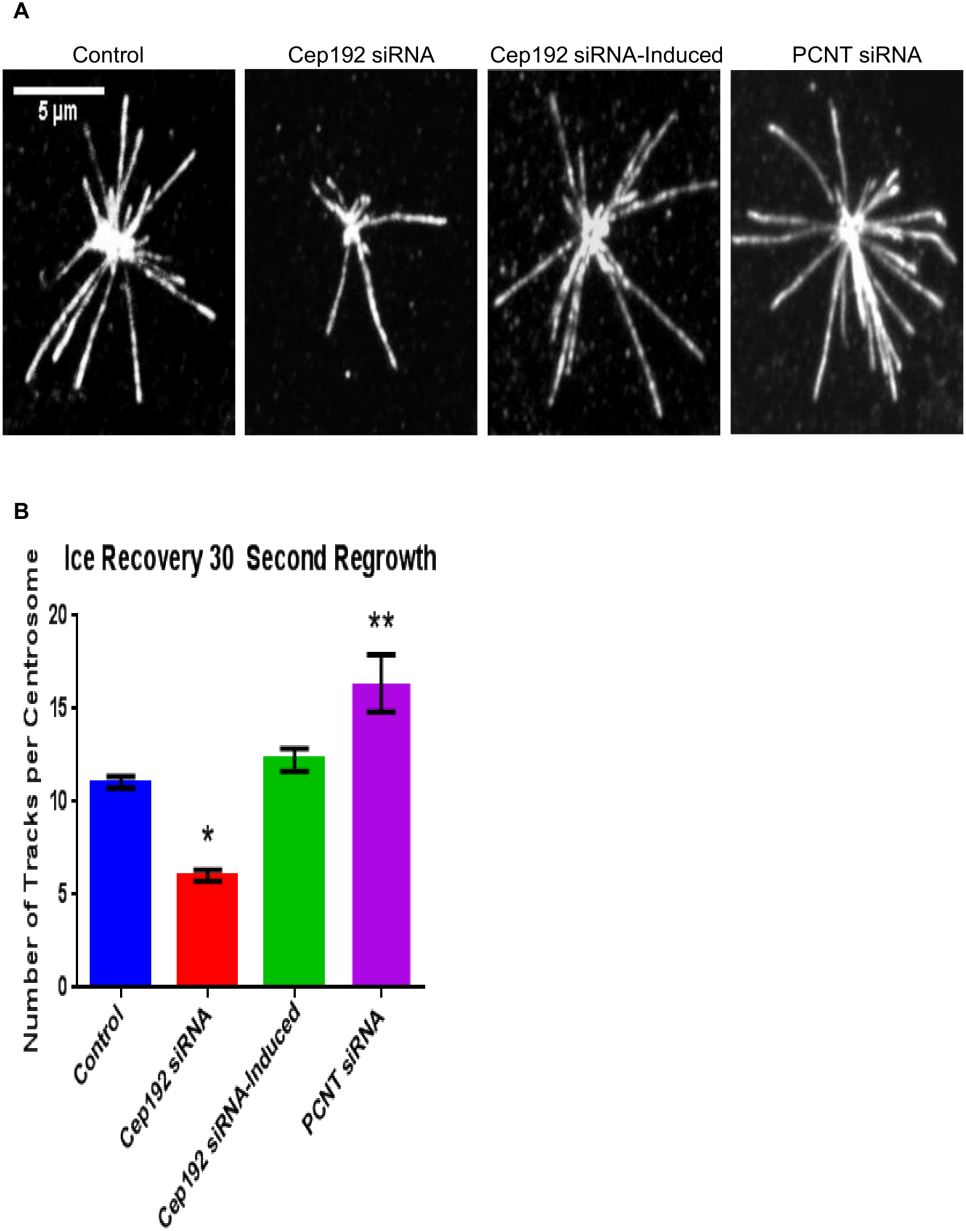
MT nucleation following Ice Depolymerization in Cep192-2 Flp-In T-Rex U2OS cells. A) Following 40 minutes of ice depolymerization, cells were incubated in 37°C media for 30 seconds to allow regrowth. Cells were fixed and stained with EB1 and Cep192. B) Following Cep192 depletion fewer MTs regrew(6.0±0.297 MTs/centrosome) than in controls (11.0±0.319 MTs/centrosome). Induction of Cep192-2 rescued the MT nucleating capacity (12.3±0.611 MTs/centrosome) of the centrosome to control levels. Depletion of Pericentrin lead to a significant increase in the number of MTs attached to the centrosome (16.4±1.54) relative to controls. Vertical bars represent S.E.M. P values are <0.001, P = 0.0691, and P = 0.0096, respectively. N≥14 cells per experiment from 3 independent experiments per condition.

The above hypothesis was tested more directly using a MT regrowth assay. The MT arrays of control and Cep192 or Pericentrin siRNA-treated U2OS cells were completely depolymerized via ice treatment for 40 minutes. Cells were then returned to room temperature for 30 seconds and fixed and double-labeled for Cep192 and EB1, as EB1 immunofluorescence has also been proven to be ideal for identifying sites of MT nucleation in similar regrowth assays [5], [34]. Using this approach we were able to identify and count the number of centrosomally nucleated MTs within the 30 second regrowth window. As expected, depletion of Cep192 from interphase cells significantly reduced centrosome MT nucleation by roughly half relative to controls–control cells contained an average of 11.04±0.319 MTs/centrosome while Cep192 siRNA-treated cells contained an average of 6.04±0.297 MTs/centrosome (Fig. 8). This phenotype was again rescued by the induced expression of FLAG-Cep192-2 (12.25±0.611 MTs/centrosome) (Fig. 8). Depletion of Pericentrin, on the other hand, had the opposite effect, significantly increasing centrosomal MT nucleation nearly 70% relative to controls and more than 300% relative to Cep192 knockdown (16.36±1.541 MTs/centrosome) (Fig. 8).

The possibility that Cep192 and Pericentrin share a similarly antagonistic relationship during mitosis seems on the surface to be somewhat unlikely, particularly given their co-dependent localization to mitotic centrosomes [11]. However, as a functional matter it is worth noting that the enrichment of Cep192 at mitotic centrosomes occurs at a level that is ∼3 to 4 fold higher than that of Pericentrin [10], [11]. Thus the relative proportion of these proteins shifts in favor of Cep192 and away from Pericentrin at the same time that the centrosome normally undergoes its largest increase in MT nucleation capacity. In our opinion, this issue is ripe for future investigation.

### Cep192 is required for cell polarization and migration

**Figure 9:**
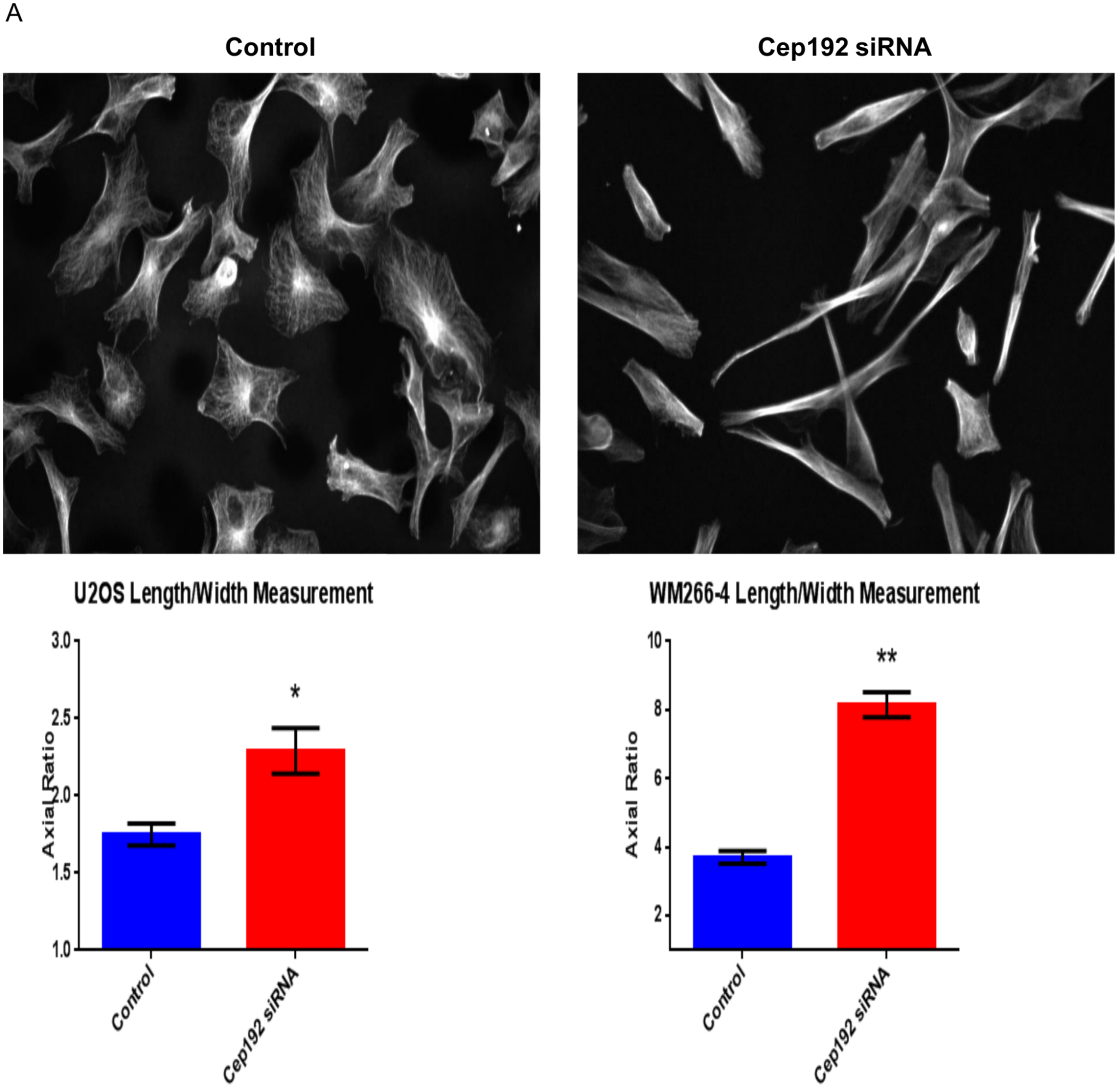
Cep192 impacts cell polarization. A) Top panels show immunofluorescence micrographs of control and Cep192 siRNA-treated WM266-4 cells stained for alpha-tubulin 72 hours after siRNA treatment. Cep192 siRNA-treated WM266-4 cells are clearly elongated relative to controls. Bottom panels show the measured average axial ratios (length/width) of control and Cep192 siRNA-treated U2OS cells and WM266-4 melanoma cells. Vertical bars represent S.E.M. P = 0.0007 (U2OS cells) and <0.0001 (WM266-4 cells). N≥20 cells from each condition per experiment from 3 independent experiments.

**Figure 10:**
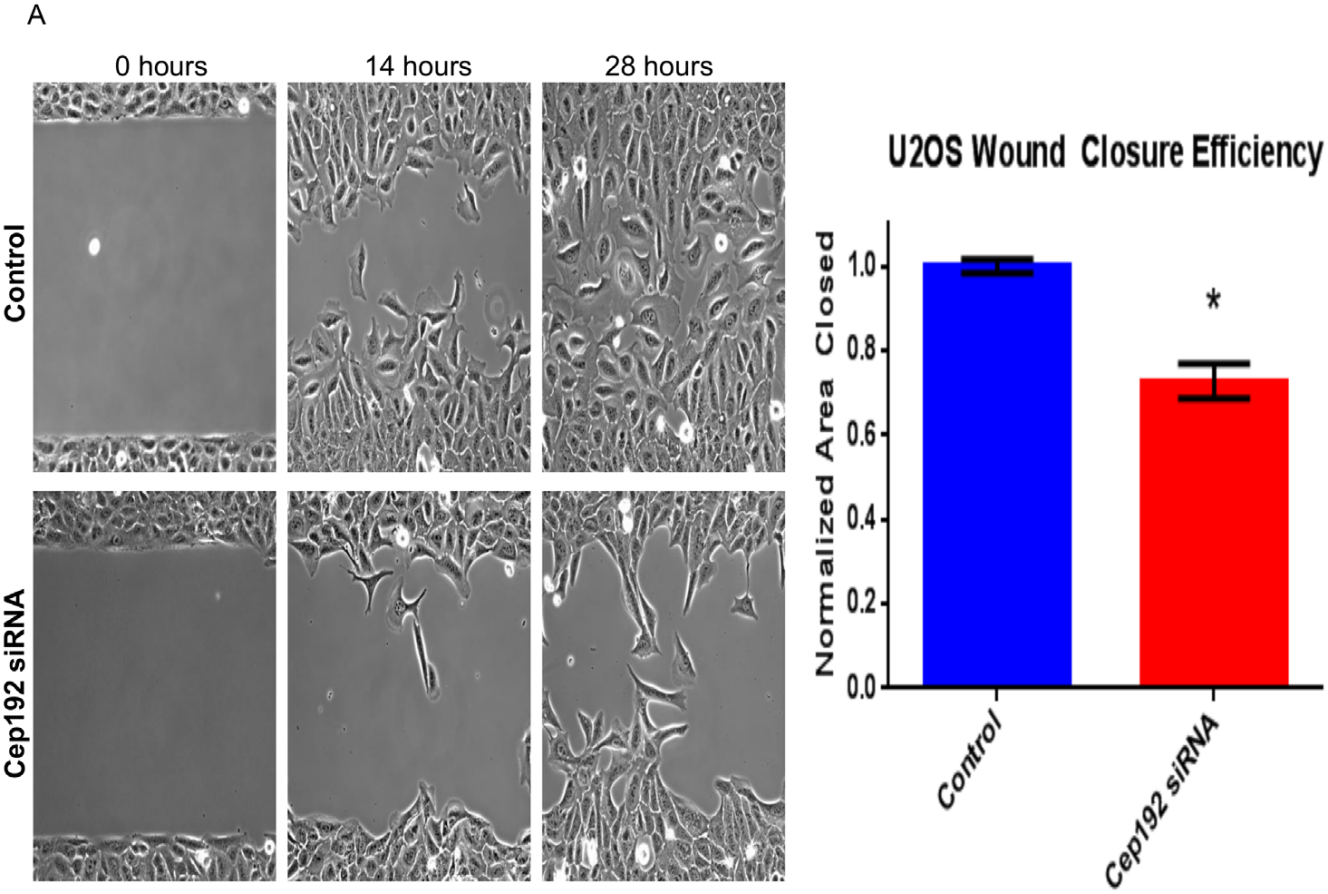
Cep192 is required for efficient polarization and cell migration. A) Phase-contrast images from a 2-D scratch assay performed on control Cep192 siRNA treated U2OS cells. U2OS cells were plated into Ibidi Culture-Insert dishes following siRNA treatment and the cell-free zone was generated by removing the insert. B) Quantification of the efficiency at which control and Cep192 siRNA-treated cells moved into the cell-free zone (determined by measuring the cell free area at the indicated timepoint). Vertical bars represent S.E.M. P<0.0001. N = 3 experiments per condition.

Finally, throughout the course of our analyses we noticed that the depletion of Cep192 caused cells to become hyperpolarized, adopting morphologies that were substantially elongated relative to controls. Consistent with this qualitative assessment, follow-up axial ratio measurements revealed a significant, ∼40% increase in the average cell length/width of Cep192 siRNA-treated U2OS cells relative to controls (Fig. 9). Other cell types showed a similar phenotype that varied by degree. For example, WM266-4 melanoma cells and primary adult human epidermal keratinocytes (HEKa) both displayed a more than 200% increase in axial ratio after Cep192 knockdown. Because cell polarization is integrally linked to cell migration, we then examined whether Cep192 knockdown affected cell motility using a standard 2-D in vitro scratch assay. Our initial hypothesis was that the loss of Cep192 and resulting increase in cell polarization would increase the rate at which cells moved. However, we found that Cep192 knockdown actually significantly reduced the rate at which U2OS cells moved into the scratch zone by ∼30% (Fig. 10). An even stronger effect was observed when similar assays performed on HEKa cells. In this case, the depletion of Cep192 reduced cell migration into the scratch zone by more than 50% (Fig. S1).

What is the basis for this effect? MTs are known regulators of cell polarization and migration at least in part through their delivery of membrane and signaling molecules to the leading edge [35]. In many cell types, cytosolic and Golgi nucleated MTs are thought to bear this task [36]. We propose that the hyperpolarization caused by Cep192 knockdown is due to 1) the loss of radial centrosomal MTs, which tend to maintain cell circularity and 2) increased directional growth of MTs from sites outside the centrosome, particularly the Golgi apparatus, which have long been linked to cellular elongation and cell polarization. The resulting imbalance of centrosome and non-centrosomal MTs inhibits the cell’s ability to dynamically interact with and alter its shape in response to environmental cues and thus attenuates constructive cell movement. Further experimentation will be required to establish the validity of this hypothesis. It will also be interesting to test whether the up- or down-regulation of Cep192 is normally utilized as a mechanism to induce changes in cell shape, for instance in epithelial cell polarization and/or neuronal process formation.

In conclusion, this study clearly identifies Cep192 as an important regulator of interphase MT arrays, particularly the nucleation of centrosomal MTs, with important roles in the establishment of cell shape and motility. Our data also strongly suggest that Cep192 is functionally antagonized by another PCM component, Pericentrin. Whether this relationship is maintained throughout the cell cycle or persists only during interphase remains to be seen. Our current functional model is that Cep192 serves to recruit gamma-tubulin to centrosomes and that this in turn helps to control the balance of centrosomal and non-centrosomal MTs. Naturally occurring and developmentally regulated shifts in this balance of MT subpopulations, perhaps stimulated by alterations in the expression of Cep192, may provide a broader mechanism for controlling cell morphogenesis and migration.

## Supporting Information

Figure S1 **HEKa wound healing assay.** A) Time-lapse phase-contrast images from a 2-D scratch assay performed on control and Cep192 siRNA treated HEKa (human epidermal keratinoctyes- adult) cells HEKa cells. B) Quantification of the number of control and Cep192 siRNA treated cells that entered the initial cell free zone at the indicated timepoints. C) Axial ratio measurements of control and Cep192 siRNA-treated HEKa cells (Control, 1.9; Cep192 KD, 3.7).
(TIF)

Figure S2 **FLAG-Cep192-2 localizes to the centrosome in Flp-In T-Rex U2OS cells.** Following 24 hours of induction with 1 ug/ml tetracycline, FLAG-Cep192**-**2 expression is induced and localizes to the centrosome.
(TIF)

Dataset S1 **Minimal datasets for experiments presented in** **Figure 1** **.**
(ZIP)

Dataset S2 **Minimal datasets for experiments presented in** **Figure 2** **–** **5** **.**
(ZIP)

Dataset S3 **Minimal datasets for experiments presented in** **Figure 6** **.**
(ZIP)

Dataset S4 **Minimal datasets for experiments presented in** **Figure 7** **–** **8** **.**
(ZIP)

Dataset S5 **Minimal datasets for experiments presented in** **Figure 9** **–** **10** **.**
(ZIP)

Movie S1 **EB1-GFP comets moving in a control U2OS cell.** Time series movies showing EB1-GFP comet movement in a control siRNA-treated U2OS cell.
(AVI)

Movie S2 **EB1-GFP comets moving in a Cep192 KD U2OS cell.** Time series movies showing EB1-GFP comet movement in a Cep192 siRNA-treated U2OS cell. Note the loss of a focused radial MT growth center after Cep192 depletion.
(AVI)
