# Supplementary figures and images for: Cep192 Controls the Balance of Centrosome and Non-Centrosomal Microtubules during Interphase
Source: PLoS One. 2014 Jun 27;9(6):e101001. doi: 10.1371/journal.pone.0101001 (PMC4074188; doi:10.1371/journal.pone.0101001)

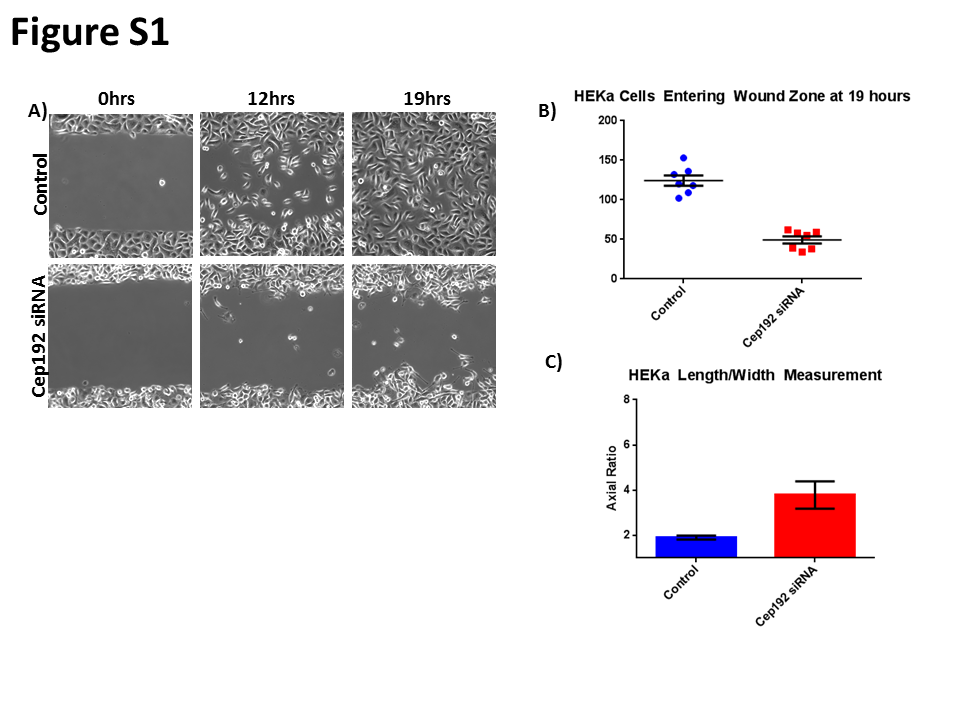

Supplement: Figure S1 — HEKa wound healing assay. A) Time-lapse phase-contrast images from a 2-D scratch assay performed on control and Cep192 siRNA treated HEKa (human epidermal keratinoctyes- adult) cells HEKa cells. B) Quantification of the number of control and Cep192 siRNA treated cells that entered the initial cell free zone at the indicated timepoints. C) Axial ratio measurements of control and Cep192 siRNA-treated HEKa cells (Control, 1.9; Cep192 KD, 3.7). (TIF) [file pone.0101001.s001.tif]

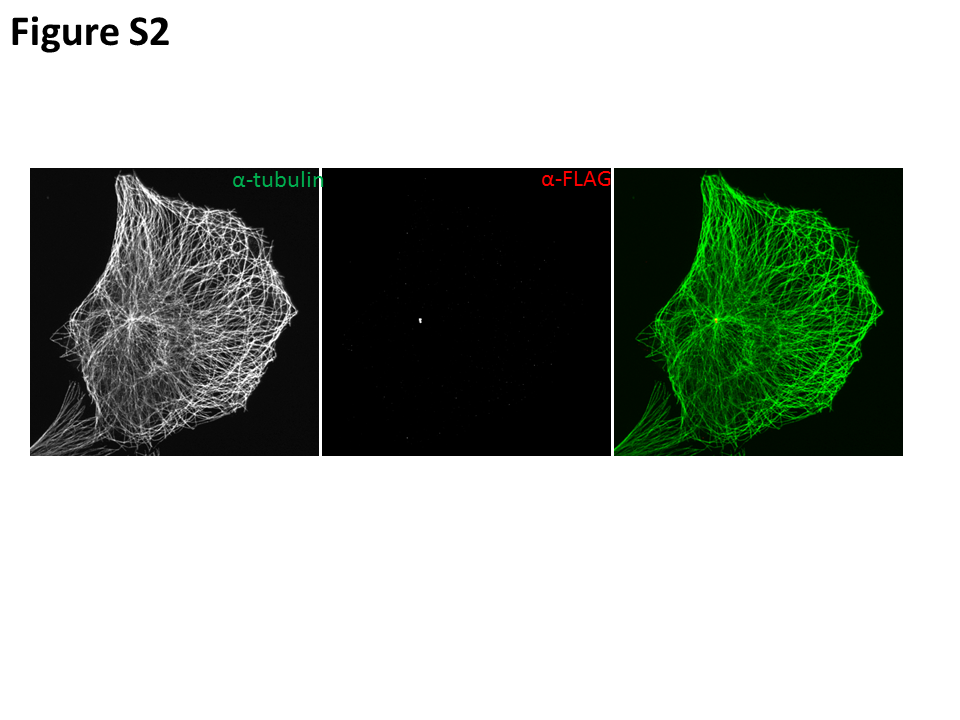

Supplement: Figure S2 — FLAG-Cep192-2 localizes to the centrosome in Flp-In T-Rex U2OS cells. Following 24 hours of induction with 1 ug/ml tetracycline, FLAG-Cep192-2 expression is induced and localizes to the centrosome. (TIF) [file pone.0101001.s002.tif]
